# Regulation of yeast polarized exocytosis by phosphoinositide lipids

**DOI:** 10.1007/s00018-024-05483-x

**Published:** 2024-11-19

**Authors:** Matthew W. Volpiana, Aleksa Nenadic, Christopher T. Beh

**Affiliations:** 1 Department of Molecular Biology and Biochemistry, Simon Fraser University, 8888 University Drive, Burnaby, BC V5A 1S6 Canada; 2 Centre for Cell Biology, Development, and Disease, Simon Fraser University, Burnaby, BC Canada

**Keywords:** Exocyst complex, Phosphoinositide kinases, Phosphoinositide phosphatases, *MSS4*, *PIK1*, PI(4,5)P_2_, PI4P, Polarized exocytosis, Post-Golgi vesicle transport, Rab GTPases, Rho GTPases, *SAC1*, *SEC2*, *STT4*

## Abstract

Phosphoinositides help steer membrane trafficking routes within eukaryotic cells. In polarized exocytosis, which targets vesicular cargo to sites of polarized growth at the plasma membrane (PM), the two phosphoinositides phosphatidylinositol 4-phosphate (PI4P) and its derivative phosphatidylinositol 4,5-bisphosphate (PI(4,5)P_2_) pave the pathway for vesicle transport from the Golgi to the PM. PI4P is a critical regulator of mechanisms that shape late Golgi membranes for vesicle biogenesis and release. Although enriched in vesicle membranes, PI4P is inexplicably removed from post-Golgi vesicles during their transit to the PM, which drives subsequent steps in exocytosis. At the PM, PI(4,5)P_2_ recruits effectors that establish polarized membrane sites for targeting the vesicular delivery of secretory cargo. The budding yeast *Saccharomyces cerevisiae* provides an elegant model to unravel the complexities of phosphoinositide regulation during polarized exocytosis. Here, we review how PI4P and PI(4,5)P_2_ promote yeast vesicle biogenesis, exocyst complex assembly and vesicle docking at polarized cortical sites, and suggest how these steps might impact related mechanisms of human disease.

## Introduction

The transport pathway for exocytosis, from post-Golgi vesicle biogenesis to vesicle targeting sites on the plasma membrane (PM), is promoted by phosphoinositide lipid gradients. Phosphoinositides are phosphorylated derivatives of phosphatidylinositol (PI) that are recognized by specific domains on proteins for their recruitment at membranes. Although distinct sets of phosphoinositides demarcate different intracellular membranes, the most relevant phosphoinositides involved in exocytosis are phosphatidylinositol 4-phosphate (PI4P) and its derivative phosphatidylinositol 4,5-bisphosphate [PI(4,5)P_2_]. In budding yeast, PI4P is mainly enriched in the Golgi and nascent vesicles that bud off of the trans-Golgi, whereas PI(4,5)P_2_ is mainly distributed at the PM and at polarized sites on the cell cortex, in a cell cycle-dependent manner [1–3]. As key regulators of polarized exocytosis, the axis of secretory transport from the Golgi to the PM is determined by PI4P and PI(4,5)P_2_ metabolism within the cell.

## Phosphoinositide metabolism lays a path for exocytosis

*Saccharomyces cerevisiae* serves as a model for understanding the regulation of phosphoinositide metabolism, as it impacts polarized exocytosis. In addition to being a well-established experimental platform for molecular genetics and, historically, a vanguard for understanding mechanisms of membrane trafficking, budding yeast is a model for investigating the dynamic regulation of membrane lipids [4–6]. In yeast, many biochemical and genetic techniques can be exploited, including the use of genetically encoded fluorescent lipid probes, some of which were first developed in mammalian systems for in vivo monitoring of lipid and phosphoinositide dynamics [7–11]. Combined, these approaches set the stage for the many extensive investigations of phosphoinositide regulatory activities during yeast polarized exocytosis.

In budding yeast, the spatiotemporal regulation of phosphoinositide metabolism is primarily dependent on the interplay between lipid kinases and phosphatases. The major synthesis of Golgi PI4P in yeast cells is conferred by the type IIIβ PI4-kinase Pik1p, whereas PI4P in the PM is produced by the IIIα PI4-kinase Stt4p [1, 12]. Lsb6p, a related type II PI4-kinase, can partially suppress the inactivation of Stt4p suggesting that Lsb6p contributes to PI4P synthesis at the PM, but Lsb6p activity is mainly associated with endolysosomal trafficking [13, 14]. In fact, unlike other fungal homologues, Lsb6p in *Aspergillus nidulans* is associated with the Golgi where it replaces the role of Pik1p in apical polarized secretion [15]. In *S. cerevisiae*, membrane lipids (including PI4P) in the Golgi are packaged into exocytic vesicles and continually transported to the PM, but PI4P pools within the Golgi and PM are distinct and do not intermix [12]. How Pik1p-generated PI4P contributes to PI(4,5)P_2_ at the PM, even though PI4P apparently ‘vanishes’ from post-Golgi secretory vesicles at the end of their transit represents an unresolved paradox [16, 17]. In yet another seeming contradiction, PI4P generated by Stt4p is largely polarized in the PM to the growing daughter cell even though Stt4p kinase is generally found in patches within mother cells [18–20]. Evidently, after PI4P synthesis, other regulatory factors control PI4P levels, cellular distribution, and dynamics within membranes.

In addition to its synthesis, PI4P metabolism is dictated by dephosphorylation as well as conversion of PI4P into polyphosphorylated phosphoinositides, such as PI(4,5)P_2_. PI4P is dephosphorylated and thereby depleted by the integral membrane protein Sac1p, which is localized in yeast to the ER under normal growth conditions [21, 22]. This localization poses a seeming contradiction given that Sac1p acts on the Stt4p-generated PI4P pool in the PM [21]. However, because Sac1p localizes to membrane contact sites between cortical ER and the PM, Sac1p activity is largely directed at the PM pool of PI4P [19, 23, 24]. Elimination of *SAC1* or the tether genes conferring ER-PM membrane contact results in uniform PI4P redistribution throughout the PM where normally PI4P is concentrated at sites of polarized growth at the PM [23, 24]. It should be noted, however, that deleting *SAC1* in cells lacking ER-PM contact sites results in lethality, indicating that Sac1p and ER-PM tethers have additional contributions beyond just maintaining PM PI4P levels [25]. In addition to Sac1p, other phosphoinositide phosphatase homologues in budding yeast appear to have wider substrate specificities for phosphoinositides than just PI4P, though they too are significant regulators of cellular PI4P [1, 21, 26]. Nonetheless, Sac1p represents an important determinant of PI4P levels and polarized distribution in the PM, which also affects the distribution of the PI4P derivative PI(4,5)P_2_ [27].

Stt4p generates PI4P in the PM, which in turn serves as the substrate for PI(4,5)P_2_ synthesis by Mss4p, the sole PI4P 5-kinase in budding yeast that is also a key determinant of polarized PM growth [18, 27–32]. Consistent with its role in controlling phosphoinositide metabolism at the PM, *MSS4* (multicopy suppressor of Stt4 mutation) was initially identified as a suppressor of the temperature-sensitive (ts) *stt4-1* mutation that affects PI4P pools in the PM [33]. However, neither *pik1*^ts^ or *stt4*^ts^ are lethal when combined with an *mss4*^ts^ allele, suggesting that the Golgi and PM pools of PI4P generated by Pik1p and Stt4p both contribute PI4P for Mss4p activity, as confirmed by the drastic reduction of PI(4,5)P_2_ in *pik1*^ts^ *stt4*.^ts^ double mutant cells [12]. In the PM, PI(4,5)P_2_ is concentrated at sites of membrane growth but synthesis of PI(4,5)P_2_ by Mss4p cannot be solely responsible for this polarized distribution; it is debatable whether PI(4,5)P_2_ has an intrinsic capability to self-form or maintain membrane domains by itself[34, 35]. Rather, the interplay between Mss4p, Sac1p, Stt4p, Pik1p, and lipid transfer proteins dynamically appear to generate localized PI(4,5)P_2_ membrane concentrations that then recruit PI(4,5)P_2_-binding effectors. PI(4,5)P_2_-enriched domains are crucial for the assembly of protein complexes exocytic vesicle targeting as the prerequisite for membrane fusion at sites of polarized growth [36–38]

In the dynamic regulation of phosphoinositide-enriched domains within membranes, soluble lipid transfer proteins play an important role in shuttling PI4P between membranes to regulate its turn-over or conversion to PI(4,5)P_2_ [39, 40]. In particular, the ORP (Oxysterol binding protein [OSBP] related protein) family is implicated in phosphoinositide lipid exchange between different membranes [41]. Yeast ORPs are encoded by the seven *OSH* (OSBP homologue) genes (*OSH1*-*OSH7*), which all share essential functions that any individual *OSH* performs in the absence of the others [42]. The canonical mammalian OSBP was first shown to change its localization in response to hydroxycholesterol binding, and ORPs were subsequently demonstrated to bind and transfer other lipids, including PI4P and PI(4,5)P_2_ [43–46]. ORPs bind sterols and phospholipids, and these lipids are exchanged for phosphoinositides, which then leads to cycles of lipid transfer between membranes. The transfer of phosphoinositides appears to be the common function of all ORPs. Given that collectively the yeast *OSH*s have shared overlapping essential function/s, it follows that *OSH*s are important regulators of the essential function of maintaining intracellular phosphoinositide distribution. Indeed, deletion of all *OSH* genes causes a marked increase in PI4P and PI4P redistribution ubiquitously around the PM [16, 23, 47]. In these cells, the disruptions in phosphoinositide regulation are concurrent with polarization defects affecting cell morphology, septin ring assembly, actin polarization, chitin deposition, and polarized exocytosis [48]. During polarized exocytosis, the archetypal yeast ORP Osh4p (also referred to as Kes1p) tracks along on post-Golgi vesicles as they transit from the Golgi to polarized sites of growth on the PM [49–51]. Moreover, Osh4p coprecipitates with regulators and subunits of the exocyst tethering complex [49]. These findings implicate Osh4p, along with other Osh proteins such as Osh6p, in the final events of vesicle targeting to the PM [51]. Indeed, the inactivation of all Osh proteins (but not removal of individual Oshs) causes the accumulation of undocked vesicles in daughter buds and a uniform redistribution of PI4P around the PM [16, 48, 49]. During vesicle biogenesis, however, *OSH4* also plays a unique role as nascent vesicles bud from the trans-Golgi on their way to the PM. *OSH4* deletion bypasses the essential requirement for *SEC14*, which encodes a PI/phosphatidylcholine (PC) transfer protein that is key for exocytic vesicle budding from the Golgi [52–55]. ORP-dependent lipid exchange helps regulate exocytosis by controlling the dynamic distribution of PI4P and PI(4,5)P_2_ [16, 50, 56], though the role in phosphoinositide regulation does not preclude additional roles for ORPs acting directly on the transport machinery.

## PI4P and DAG promote post-Golgi vesicle biogenesis

**Fig. 1:**
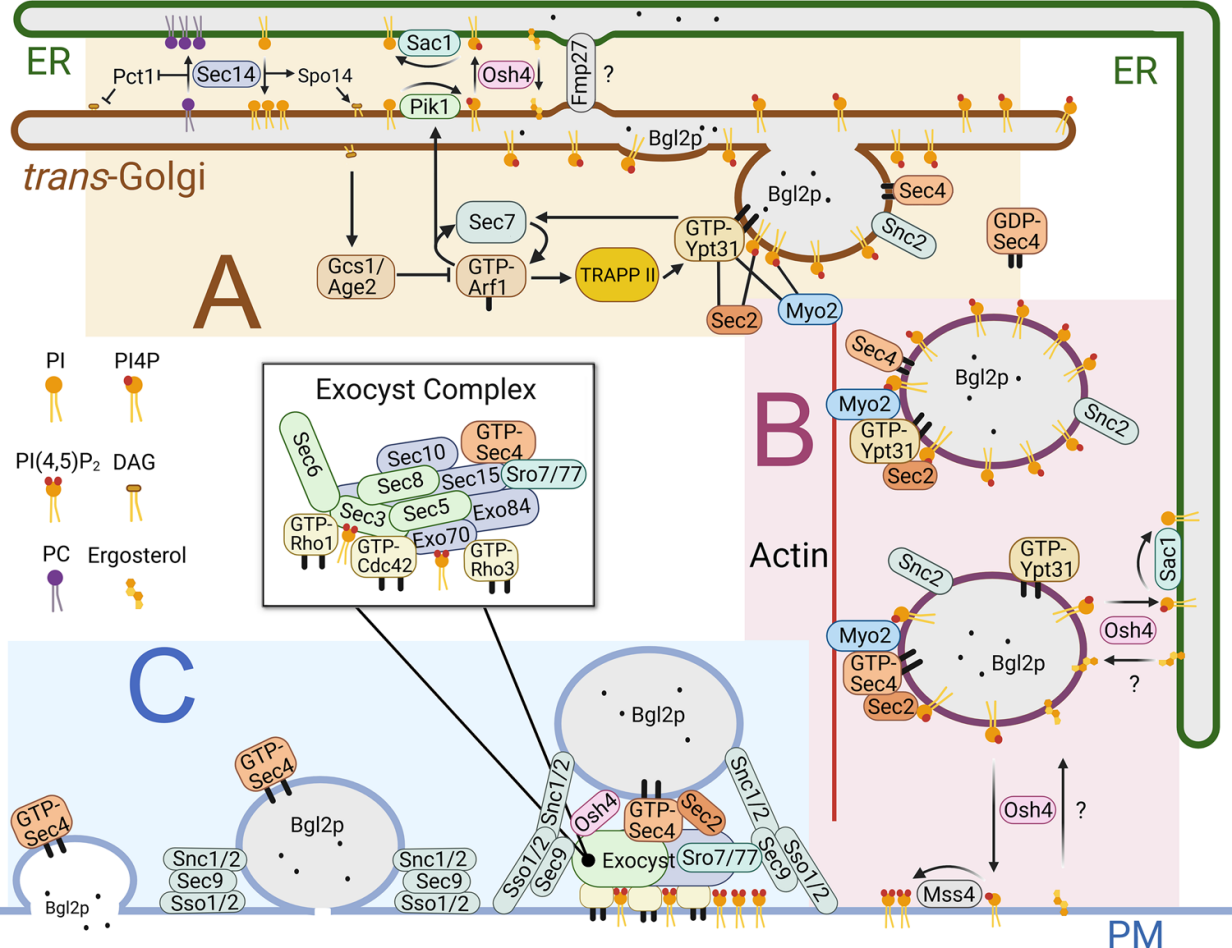
Phosphoinositide regulation of polarized exocytosis from the Golgi to the PM. **(A)** During Golgi vesicle biogenesis, the balanced exchange of lipids between the ER and Golgi lays the groundwork for PI4P generation. Sec14p reciprocally transfers PC from the trans-Golgi to the ER and PI from the ER to the trans-Golgi. PC-bound Sec14p inhibits DAG production through Pct1p whereas PI-bound Sec14p promotes DAG production via Spo14p. In the trans-Golgi, the Pik1p kinase converts the newly transferred PI to PI4P. In the opposing direction, Osh4p removes Golgi PI4P by transferring PI4P to the ER, where the Sac1p phosphatase dephosphorylates PI4P to PI. Golgi PI4P recruits Rab GTPases Ypt31/32p and indirectly stimulates Sec7p GEF activity, which in turn stimulates Arf1p GTPase activity. Arf1p activates the TRAPPII GEF complex and the Pik1p kinase, which further amplifies PI4P production and Ypt31/32p activity. Golgi accumulation of DAG stimulates Gcs1p and Age2p two Arf GAPs that inactivate Arf1p during later stages of vesicle development. **(B)** As vesicles are released from Golgi, Ypt31p/32p and PI4P recruit the Rab GEF Sec2p and the type V myosin motor Myo2p for vesicle transport along actomyosin cables towards sites of polarized membrane growth. As a mechanism for Sec2p activation of the Rab GTPase Sec4p, PI4P in the vesicle membrane is removed during vesicle transit. Osh proteins (e.g. Osh4p) facilitate PI4P removal from vesicles either by vesicle-ER lipid exchange, in which Sac1p turns-over PI4P in the ER, or vesicle-PM exchange, wherein PI4P is converted to PI(4,5)P_2_ by Mss4p in the PM. **(C)** PI4P removal from vesicles causes Sec2p to swap Ypt31p/32p for Sec4p binding and activation. Sec2p and GTP-Sec4p recruit Sec15p for the assembly of the exocyst complex. On the PM, PI(4,5)P_2_ clusters the Rho GTPases Cdc42p, Rho1p and Rho3p. During vesicle docking with the PM, the exocyst complex subunits Sec3p and Exo70p bind PI(4,5)P_2_ and the Rho GTPases. As mediated by Sec6p and Sro7p/77p, tethering by the exocyst complex transitions to membrane fusion as mediated by the Snc1p/2p v-SNAREs and Sso1/2p, Sec9p t-SNAREs

After protein translocation into the ER and the subsequent delivery of secretory proteins to the late Golgi, the Pik1p-dependent synthesis of PI4P regulates vesicle biogenesis that initiates exocytic trafficking to the PM [12, 21, 53] (Fig. 1A). The production of PI4P by Pik1p is limited by the synthetic availability of its substrates, which is contingent on PI (phosphatidylinositol) transfer from ER to the Golgi [53]. As a PI/phosphatidylcholine (PC) transfer protein, Sec14p is proposed to move PI precursors for PI4P synthesis in the Golgi, which is ultimately essential for post-Golgi vesicle biogenesis [53, 54]. Inactivating Sec14p PI/PC transfer function has the same effect on vesicle biogenesis as eliminating Pik1p [53, 55, 57, 58]. Moreover, Pik1p overexpression in *sec14-3* cells can partially rescue growth and secretion defects, but Sec14p overexpression is unable to rescue growth defects of *pik1-63* or *pik1-83* cells [53]. Thus, these results are consistent with the concept that Sec14 acts upstream to regulate Pik1p generation of Golgi PI4P. Despite that Sec14p transfers PI and PC between liposomal membranes in vitro, mutations that impair Sec14p binding to PI or PC do not disrupt its essential in vivo functions during post-Golgi secretion [58–60]. These results suggest that while Sec14p lipid transfer activity may contribute to its overall physiological function, Sec14p impacts vesicle formation as a regulator of phospholipid metabolism and PI4P synthesis in Golgi compartments.

The regulatory role of Sec14p during PI4P-dependent vesicle biogenesis has been revealed through the identification of suppressors that bypass the essential function of *SEC14* [47, 61–64]. The cellular requirement for *SEC14* can be entirely abolished by inactivating specific genes encoding phospholipid synthetic enzymes or, through a mechanism affecting Pik1p-generated PI4P, by deleting *SAC1* or *OSH4*/*KES1* [61–64]. These findings suggest that Sec14p links PI4P regulation and phospholipid synthesis. PI-bound Sec14p increases Pik1p-dependent production of PI4P and PI(4,5)P_2_, and thereby activates Spo14p, a phospholipase D homologue that converts PC to phosphatidic acid (PA) for DAG synthesis [65, 66]. On the other hand, PC-bound Sec14p inhibits choline-phosphate cytidylyltransferase thereby inhibiting PC synthesis and increasing DAG pools [67]. Through both mechanisms, Sec14p activity increases DAG within the Golgi membrane bilayer, which is proposed to affect vesicle biogenesis via membrane deformation and curvature, as well as inducing membrane disorder promoting vesicle scission [68–70]. Others, however, question the overall impact of DAG on Golgi membrane curvature [71]. It is curious that Pah1p, the PA phosphatase that produces DAG, has no reported role in the formation of exocytic vesicles from the late Golgi. Regardless, DAG acts in other ways to activate vesicle biogenesis at the trans-Golgi, alongside PI4P [69, 72].

DAG and PI4P metabolism cooperate in the activation of Arf1p (ADP-ribosylation factor 1), a small GTPase regulator of Golgi maturation and post-Golgi vesicle formation [73] (Fig. 1A). Early in the process in vesicle formation, Arf1p is activated by the guanine nucleotide exchange factor (GEF) Sec7p, which acts to recruit more GTP-bound Arf1p to the Golgi membrane thereby creating a positive-feedback loop [74]. Moreover, Sec7p is also an effector of Golgi Rab GTPases including Ypt31p/Ypt32p, which are critical for the regulation and recruitment of later effectors of post-Golgi vesicle transport [75, 76]. Ypt31p/Ypt32p further augments Sec7p GEF activity that, together with increased PI4P levels, drives cargo sorting into vesicles [72, 75]. Sec7p activation of Arf1p also recruits Pik1p to further boost PI4P production; inhibition of either Arf1p or Sec7p results in Pik1p mislocalization and impaired Golgi PI4P production [12, 72, 77]. As shown by in vitro liposome binding, DAG also facilitates GTP-Arf1p recruitment of Pik1p, through in vivo the role of DAG is less clear [77]. In yeast cells, DAG appears to promote vesicle release from the Golgi by stimulating Gcs1p and Age2p ArfGAP activities, which in turn leads to membrane dissociation of GDP-bound Arf1p [78, 79]. In this manner, DAG facilitates Arf1p GTP-to-GDP switching for vesicle biogenesis, enabling Arf1p recycling after membrane scission for subsequent rounds of vesicle formation.

## The balancing act controlling Golgi PI4P

As bypass suppressors of *SEC14*, the deletions of *SAC1* and *OSH4* appear to impact PI4P incorporation into post-Golgi vesicles by limiting PI4P accumulation within the trans-Golgi [80] (Fig. 1A). Under nutrient-plentiful conditions, yeast Sac1p resides in the ER close to ER-PM contact sites where it primarily affects the Stt4p-synthesized PI4P pool within the PM [25, 81]. Given that Sac1p localization is mainly restricted to the ER, Sac1p lacks a direct means to access Golgi PI4P. Current models involve PI4P transfer from the Golgi to the ER, where PI4P is then hydrolyzed to PI by Sac1p [26, 44, 53]. In mammalian cells, OSBP is recruited to ER-Golgi membrane contact sites (MCSs) where it transfers ER-derived cholesterol to the Golgi and then returns Golgi-synthesized PI4P to the ER, where it is presented to Sac1p [41, 82, 83]. OSBP-dependent increases of cholesterol in the Golgi recruits PI 4-kinases, which represents one mechanism leading to local increases in PI4P [84, 85]. In this manner, OSBP controls the balance between Golgi PI4P synthesis and its turn-over by Sac1 in the ER. Interactions between the OSBP PH (Pleckstrin Homology) domain and Golgi PI4P, and between the OSBP FFAT motif and the ER tether VAP-A, direct OSBP localization to ER-Golgi MCSs [86]. Although the function of yeast Osh4p approximates that of OSBP, Osh4p lacks the sequence elements that confer OSBP recruitment to mammalian ER-Golgi MCSs [39, 41]. However, if ER-Golgi MCSs facilitate Osh4p-dependent PI4P regulation at the trans-Golgi, then elimination of ER-Golgi tethers might also bypass the essential function of *SEC14*, like *SAC1* or *OSH4* deletions. Although no known tethers have been reported as *SEC14* bypass suppressors, the putative ER-Golgi tether/lipid transfer protein Fmp27 must be present to permit the bypass suppression of *sec14*∆ when *OSH4* or phosphatidylcholine biosynthetic genes are deleted [63]. *FMP27* encodes a Vps13p-like tether/lipid transfer protein that localizes to multiple MCSs [87] and might be a candidate tether that links the ER and Golgi together to promote Osh4p lipid transport. Regardless, Osh4p and Sac1p act on Golgi PI4P as a brake to control Sec14p stimulation of Pik1p-dependent PI4P synthesis.

## PI4P sorts lipids and proteins during post-Golgi vesicle biogenesis

The accumulation of PI4P in the Golgi leads to local changes in lipids, secretory cargo sorting, and membrane coat formation that elicit exocytic vesicle biogenesis. In addition, membrane distortions generate bilayer curvature to initiate vesicle budding from the Golgi. Drs2p is a type IV P-type ATPase “lipid flippase” that translocates phosphatidylserine (PS) and phosphatidylethanolamine (PE) across the membrane bilayer to the cytoplasmic leaflet and establishes membrane asymmetry in the trans-Golgi [88, 89]. Drs2p flippase activity is activated by PI4P [90, 91], and Drs2p-generated membrane subdomains primes membrane curvature for shaping into exocytic vesicles by coat proteins [89, 92–95]. Through Drs2p flippase activity, clathrin facilitates the formation of post-Golgi vesicles and aides exocytic cargo sorting [89, 92, 94]. Drs2p-dependent PS leaflet flipping is activated by Pik1p, and Osh4p action opposes Pik1p, therefore Drs2p and Osh4p have mutually antagonistic activities [89–91, 96]. In addition, Osh4p-dependent depletion of Golgi PI4P reduces Drs2p activity [89–91, 96]. The dynamic equilibrium of Golgi lipid composition as controlled by Drs2p and Osh4p generates the membrane architecture imparted by the lipids found in exocytic vesicles budding from the Golgi [97].

As a downstream consequence of Pik1p and Sec14p activities, with membrane conditions set by lipid recruitment and PI4P signaling, protein sorting and cargo accumulation is dictated by Arf1p regulation of clathrin and exomer adapter complexes [54, 66, 98, 99] (Fig. 1A). The direct (endosome-independent) route to the PM is defined by lower-density post-Golgi vesicles containing the secreted endoglucanase Bgl2p, which is a marker for polarized exocytosis to sites of membrane growth in daughter buds [100]. Bgl2p polarized exocytosis is dependent on Sec14p and Osh proteins where the polarized targeting of vesicular traffic to regions of active growth ensures the accumulation of protein cargo necessary for surface expansion [48, 54, 100]. In addition to the sorting of Bgl2p with other cargo destined for polarized sites, nascent vesicles are loaded with the transport machinery necessary for post-Golgi vesicle transit, docking and fusion with the PM [101]. The coordination of the next post-Golgi steps in exocytic vesicle trafficking are also regulated by phosphoinositides, which affect the sequential activation of several different small GTPases whose activities control specific transport events [17, 102, 103]. The Pik1p-dependent generation of PI4P induced by Sec7p, Arf1p, and DAG ensures recruitment of factors into post-Golgi vesicles needed later during vesicle transport to the PM. Sec7p activation of Arf1p recruits the TRAPPII GEF complex to the trans-Golgi to induce the Ypt31/32p Rab GTPases [2, 75, 104]. GTP-Ypt31/32p further activates Sec7p, which along with Pik1p-generated PI4P, leads to the assembly of essential factors for the final steps of vesicle budding from the trans-Golgi [2, 105]. At sites of vesicle budding, GTP-Ypt31/32p and PI4P recruit the Rab GEF Sec2p, a crucial regulator of later stages in exocytosis [106–108]. Sec2p is not a GEF for Ypt32p, rather the Sec2p-Ypt32p interaction only serves to attach Sec2p, along with its binding to PI4P, to nascent post-Golgi vesicles [107, 108]. The Sec2p GEF is, however, required for Sec4p Rab GTPase activation that designates the next event in the regulatory cascade of small GTPases during polarized exocytosis [107] (Fig. 1B). On nascent vesicles, Sec4p, Ypt31/32p, and PI4P also bind the type V myosin Myo2p to initiate the general movement of exocytic vesicles along actin cables to sites of polarized growth on the PM, as detailed below [109–111].

## PI4P facilitates vesicle maturation during transit and along actin/myosin cables

In addition to directing post-Golgi vesicle transit to the PM, Myo2p affects the final mechanical steps in vesicle release from the trans-Golgi (Fig. 1B). Myo2p can induce membrane curvature and vesicle budding by pulling on the membrane [112], though some vesicles appear to be released from the Golgi without Myo2p action [113]. The initial Myo2p-dependent attachment of exocytic vesicles, and their continued association with actin cables, appears to be at least partly dependent on PI4P incorporation into vesicle membranes. Sec4p and Ypt31/32p GTPases act together to bind vesicles to myosin-associated actin transport cables, but the necessity for Myo2p-Rab GTPase interactions can be bypassed by enhancing Myo2p interaction with PI4P [110]. Consistent with these results, increasing Golgi PI4P using *sac1*Δ or *PIK1* overexpression rescues the defective localization of myo2-12p at its restrictive temperature, underscoring the importance of PI4P in Myo2p localization [110]. In the timeline of exocytic vesicle transit, Ypt31/32p remains on vesicles after budding from the Golgi whereas both Sec2p and Sec4p co-localizes with Ypt31/32p immediately prior to vesicle release [113]. In close succession, Myo2p localizes on budding vesicles after Sec4p, suggesting that Myo2p is immediately recruited upon Sec4p activation [113]. In addition to the Rab GTPases and PI4P interactions with Myo2p, Myo2p also interacts with Sec15p, a subunit of the exocyst tethering complex that establishes first contact between exocytic vesicles and the PM [111, 114]. The current model envisages Ypt31/32p first capturing Myo2p as vesicles bud from the trans-Golgi after which Ypt31/32p is swapped with GTP-Sec4p and Sec15p, which both bind Myo2p until transport nears completion [111]. Through the interaction with Sec15p, Myo2p indirectly binds and affects other exocyst complex components, perhaps facilitating the assembly of the exocyst tether complex.

## PI4P regulation of Sec2p is the key spatiotemporal regulator of exocyst complex assembly

**Fig. 2:**
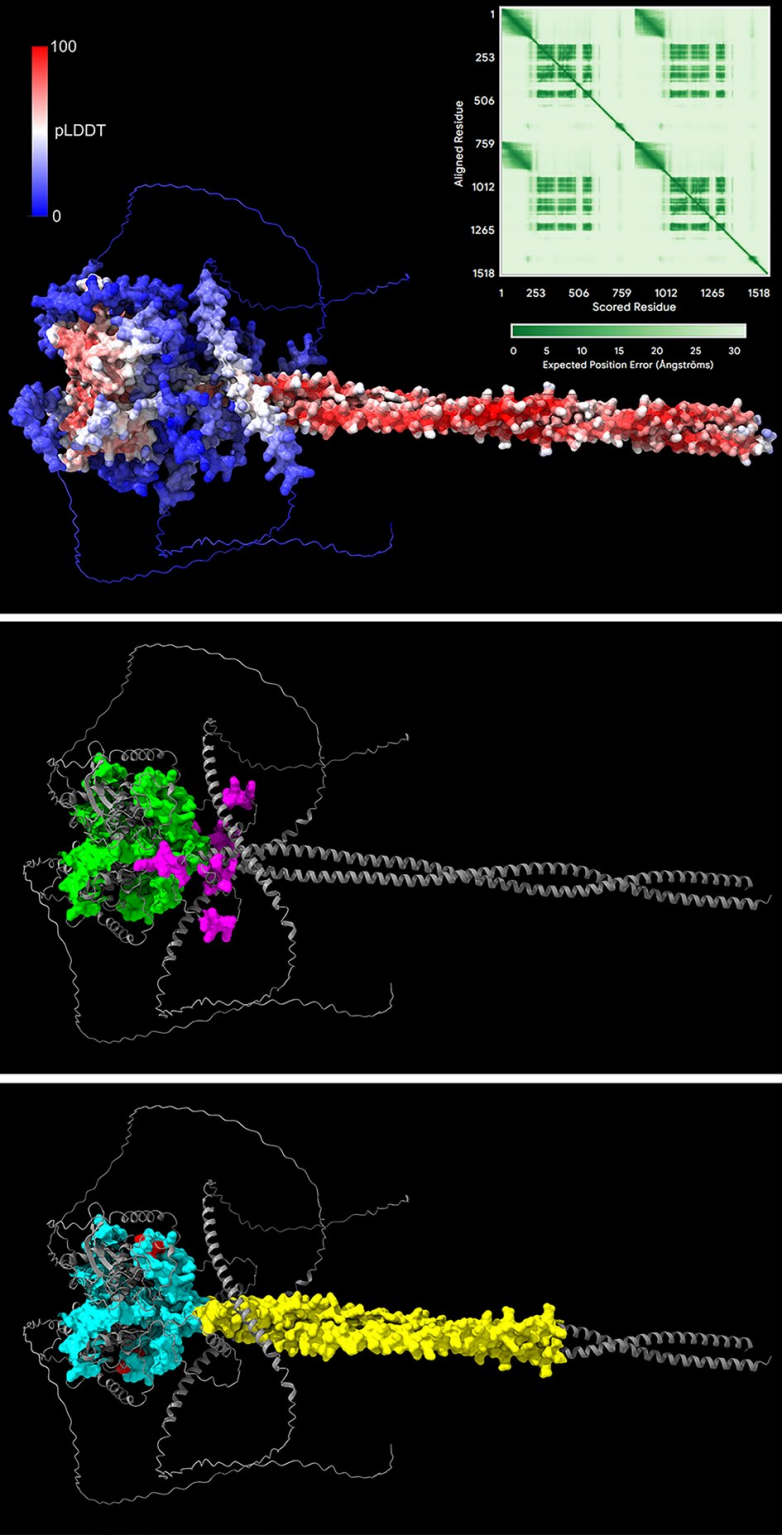
AlphaFold3-predicted Sec2p domains [242] involved in regulator and effector binding. (**Top**) Predicted structure of homodimeric full-length Sec2p shown with colouring corresponding to predicted local distance difference test (pLDDT) per-residue confidence scores; disordered C-terminal regions shown as simple lines. Residue-residue alignment confidence is shown for the Sec2p homodimer in the predicted aligned error (PAE) plot. (**Middle**) Sec2p domain interactions during vesicle biogenesis. Sec2p(160–258) region (green) binding Ypt31/32p and Sec2p(421–456) basic patches (magenta) binding PI4P. Binding domains shown as space-filling representations and other regions shown as ribbon diagrams. (**Bottom**) Sec2p domain interactions during Sec4p activation and initiation of exocyst complex assembly. Sec2p(60–160) region (yellow) binding Sec4p, Sec2p(160–258) region (light blue) binding Sec15p, and Sec2p(S181, S186, S188) phosphorylated serine residues (red). Binding domains shown as space-filling representations and other regions shown as ribbon diagrams. The Sec2p PI4P-binding and phosphorylation sites are positioned between the Sec2p GEF (Sec4p interaction) helical domain and the “regulatory” (Ypt31p/32p and Sec15p interaction) globular domain

One of the most consequential effects of PI4P on post-Golgi vesicle trafficking to the PM involves the recruitment and regulation of the Sec2p GEF. During vesicle budding from the Golgi, Sec2p binds Ypt31p/32p and PI4P on nascent post-Golgi vesicles [106–108] (Fig. 2, Middle). Sec2p has three low-affinity PI4P binding sites that appear to play a direct role in regulating Sec2p activation of small GTPases and effector interactions [108]. These PI4P binding sites map to three positively charged patches inside predicted loops region towards the C-terminal end of Sec2p, and the PI4P-binding patches are collectively required for Sec2p localization to vesicles [108] (Fig. 2, Middle). Sec2p is mislocalized in *pik1-101* cells at their restrictive temperature, confirming the dependence of Sec2p recruitment on Golgi PI4P production [108]. Due to the relatively low specificity of these charged patches, Sec2p can be recruited by other negatively charged lipids, albeit to a lesser extent [108]. Although PI4P and Ypt31/32p recruit Sec2p to vesicles, PI4P does not affect the binding of Ypt31/32p to Sec2p [108]. Thus, Sec2p recruitment to vesicles involves co-incidence detection of both Ypt31/32p and PI4P.

Ypt31/32p and PI4P recruit Sec2p to nascent vesicles, but these interactions inhibit the GEF function of Sec2p during the activation of the Sec4p small GTPase, which is a key regulator of later exocytic events [108] (Fig.1B). Sec2p does not act as a GEF for the Ypt31/32p small GTPases, despite their physical interaction [107]. Rather, Ypt31/32p binding to Sec2p adjacent to the Sec2p GEF domain blocks Sec15p binding [108, 115] (Fig. 2, Bottom). 1 The transition of Sec2p binding of Ypt31/32p to the GEF activation of Sec4p and Sec2p-Sec15p binding is attendant with the removal of PI4P from the transiting vesicles and from Sec2p itself [102, 108].

Ypt31/32p and PI4P not only affects Sec2p GEF activation of Sec4p, but they also inhibit Sec2p interactions with Sec15p, a component of the exocyst complex recruited by GTP-Sec4p [26, 108, 116]. Ypt31/32p competes with Sec15p for binding to the same domain of Sec2p [108, 117] (Fig. 2). Upon depletion of PI4P from post-Golgi vesicles, the Sec2p GEF activates Sec4p binding and favours Sec15p binding over Ypt31/32p. After PI4P removal, the complex of GTP-Sec4p, Sec15p, and Sec2p then elicits the initial events of exocyst complex assembly [108, 113] (Fig. 1C). In the octameric exocyst complex, Sec15p forms a tetrameric subcomplex helical bundle with Sec10p, Exo70p and Exo84p [116]. The Sec15p nucleated subcomplex then makes extensive interfaces with the other exocyst tetrameric subcomplex, formed by Sec3p-Sec5p-Sec6p-Sec8p, which generates the holo-exocyst complex. Given its length, Sec15p has a particularly important role as a scaffold to unite subcomplexes on vesicles, which affects the assembly of the entire exocyst complex [116]. In concert with the small GTPases Rho1p, Rho3p and Cdc42p, and the lethal giant-larvae homologues Sro7/77p at the PM, the exocyst complex then tethers vesicles to the PM and permits ensuing SNARE-mediated membrane fusion [108, 113, 118] (Fig. 1C). Thus, as the trigger for Sec2p regulatory activity, reduction of PI4P levels from post-Golgi vesicles is key to initializing exocyst complex docking upon vesicle arrival at the PM.

To explain how PI4P might be depleted from vesicle membranes while en route to the PM, it was proposed that the PI4P is removed by Osh proteins in their capacity as soluble phosphoinositide lipid transfer proteins [39, 108] (Fig. 1B). The removal of PI4P from vesicles, and PI4P dissociation from Sec2p, promotes Sec2p phosphorylation by the casein kinases Yck1p/2p thereby triggering Sec2p-Sec4p-Sec15p binding, and exocyst complex assembly [108, 117]. The Yck1p/2p phosphorylation sites lie within the Sec2p domain defined by the mutually exclusive binding of Ypt32p and Sec15p [117] (Fig. 1). The Yck2p kinase binds the Sec2p autoinhibitory domain adjacent to its PI4P binding pockets; PI4P binding inhibits Sec2p phosphorylation [117]. The proposed role of Osh4p in removing PI4P from post-Golgi vesicles, and thereby from Sec2p, is consistent with the finding that *OSH4* deletion inhibits Sec2p-Sec15p binding [39, 50, 117]. Furthermore, the combined deletions of both *YCK1* and *OSH4* cause severe growth defects in keeping with functional interaction between Osh4p and Sec2p phosphorylation [119]. Thus, PI4P extraction from the exocytic vesicles appears to be an essential event for the final steps of exocytosis at the PM.

## Osh proteins deplete PI4P from the vesicle membrane to promote exocyst complex assembly

In addition to unique affinities for either sterols or PS, all Osh proteins appear to bind phosphoinositides, and collectively they are required for phosphoinositide metabolism and regulation of polarized secretion [16, 20, 23, 39, 49, 51, 120]. Nonetheless, Osh4p remains the focus of study for determining Osh protein activities during vesicle-PM tethering, which is distinct from the unique role of Osh4p during Sec14p-dependent Golgi vesicle biogenesis [48, 49] (Fig. 1). After vesicle biogenesis, Osh4p remains on post-Golgi vesicles during their entire transit to the PM [49–51]. Osh4p co-precipitates with multiple subunits of the completely assembled exocyst complex, including the PM- and vesicle- associated small GTPases Cdc42p, Rho1p, and Sec4p [49]. Moreover, in cells where Osh proteins have been inactivated, Cdc42p and Rho1p polarization on the PM is disrupted and Sec4p accumulates on undocked exocytic vesicles [48]. Multicopy *OSH4* suppresses *CDC42* mutations that specifically affect polarized exocytosis and *OSH4* overexpression re-establishes the polarized localization of the corresponding mutant Cdc42 proteins [48]. These findings suggested that through their lipid exchange activities, Osh4p and other Osh proteins remove PI4P from vesicles and thereby promote exocyst complex tethering during vesicle docking with the PM [39].

Given that Osh4p transfers PI4P and binds to the entire assembled exocyst complex, two possibilities are suggested: (1) Osh4p is recruited to nascent secretory vesicles by PI4P and exocyst complex subunits where, as a soluble lipid transfer protein, Osh4p removes and transports PI4P from post-Golgi vesicles to the ER for PI4P dephosphorylation by Sac1p [39, 50]; (2) Osh4p is recruited to the vesicle by PI4P and exocyst complex subunits where Osh4p transfers PI4P from vesicles to the PM for PI4P conversion to PI(4,5)P_2_ by the PI4P-5-kinase Mss4p (Fig. 1B). In either case, Osh4p physical association with components on the assembled exocyst complex suggests that vesicular PI4P depletion by Osh4p does not initiate but rather maintains exocyst complex assembly. Even though Osh4p is continuously associated with transiting post-Golgi vesicles, PI4P dissipates from vesicles only when in the vicinity of the PM [16, 49–51]. Consistent with findings that myosin association with vesicles is partially dependent on vesicle PI4P, PI4P removal at the end of exocytosis is concomitant with Myo2p detachment from vesicles upon arrival at the PM. In addition to how Osh proteins affect vesicular PI4P, Osh proteins also impact PI(4,5)P_2_ levels on the PM given that PI(4,5)P_2_ levels are reduced in cells following Osh protein inactivation [23, 56]. It is not clear whether Osh proteins somehow make the Pik1p Golgi/vesicle pool of PI4P available for PI(4,5)P_2_ production by Mss4p. During vesicle docking at the PM, the close proximity of two membranes might facilitate Osh4p sterol/PI4P exchange thereby providing PI4P to fuel Mss4p production of PI(4,5)P_2_ production in the PM. Nonetheless, because Osh4p removes PI4P from vesicles it is a key driver in maintaining exocyst complex stability, which involves continued activation of Sec4p by its GEF Sec2p.

## PI(4,5)P_2_ at sites of polarized growth in the PM recruits regulators of exocytic vesicle docking and membrane fusion

In both yeast and metazoan cells, the final target site for polarized exocytosis on the PM is delimited by PI(4,5)P_2_-enriched areas where exocytic vesicles dock prior to membrane fusion and cargo delivery [38, 121, 122]. On the PM, the generation of PI(4,5)P_2_ by Mss4p affects the targeting of post-Golgi vesicles by affecting the polarized positioning of actin cables, which determines the direction of vesicle transit, and by amassing small GTPases on the PM to enable exocyst complex tethering [29] (Fig. 1C). Secretory vesicles are transported along actin cables near sites of polarized cell growth at the PM; disruption of actomyosin cables inhibits polarized exocytosis and causes PM defects that lead to isotropic cellular growth [3, 12, 21, 29, 123–125]. The Rho-like small GTPase Cdc42p interacts with upstream and downstream effectors to nucleate the actin cytoskeleton and, in turn, polarized exocytosis delivers Cdc42p to the PM to maintain membrane polarization [28, 126, 127]. The main Cdc42p GEF, Cdc24p, establishes cell polarization that ultimately promotes polarized exocytosis. However, due to its low affinity phosphoinositide-binding PH domain, the role of Cdc24p in polarized exocytosis is largely PI(4,5)P_2_-independent [128]. Other PI(4,5)P_2_-binding effectors such as Gic1p/2p, however, bind PI(4,5)P_2_ and activate Cdc42p during polarized exocytosis and polarized growth [126, 129–131]. In addition to Cdc42p, Rho3p plays dual roles in both the polarized positioning of the actomyosin cytoskeleton and in exocyst complex tethering; Rho3p directly interacts with Myo2p and the exocyst complex component Exo70p[132, 133]. Exo70p binds both Rho3p and PI(4,5)P_2_, like Cdc42p effectors that bind PI(4,5)P_2_ [134]. The third Rho GTPase involved in exocyst complex tethering to the PM, Rho1p, is also directed to polarized membrane sites by PI(4,5)P_2_ [135]. While Cdc42p is important in establishing cellular polarization, Rho1p and Rho3p appear to be necessary for its maintenance. Although Rho1p signaling controls actin organization, direct physical interaction between Rho1p and the actomyosin cytoskeleton has not been reported [136]. Nonetheless, PI(4,5)P_2_ acts alongside Cdc42p, Rho1p, and Rho3p to interact with exocyst complex components to receive vesicles at sites of polarization on the PM.

Rho GTPases ensure proper targeting of post-Golgi vesicles through direct interactions with specific “landmark” exocyst complex components, though vesicle docking is further augmented by PI(4,5)P_2_ interaction [30, 118, 137–142] (Fig.1C). As landmark exocyst complex subunits, Sec3p directly binds Rho1p whereas Exo70p interacts with both Cdc42p and Rho3p [30, 143]. The Sec3p PH domain and polybasic surface patches at the C-terminus of Exo70p also enhance association with the PM via PI(4,5)P_2_ binding [30, 38, 144]. Indeed, increased PM PI(4,5)P_2_ production by *MSS4* kinase overexpression can rescue growth defects of *SEC3* and *EXO70* mutants [3, 30, 144]. The metazoan Exo84p homologue interacts with phosphoinositides through an atypical PH domain. However, yeast Exo84p does not contain a PH domain and PI(4,5)P_2_ interactions with the yeast exocyst complex at the PM appears to be restricted to Sec3p and Exo70p [145, 146].

Unlike most yeast exocyst complex subunits, interactions between the mammalian subunits are more dynamic given that components neither arrive concurrently nor remain together at exocytic fusion sites [147, 148]. However, even in yeast there is some question of when and where some components assemble with the entire exocyst complex. Yeast Sec3p and Exo70p can be targeted to the PM independently of actin/Myo2p-mediated vesicle transport, but Sec3p and Exo70p appear to be stably associated with the entire octameric exocyst complex during vesicle transit to the PM [144, 149, 150]. Adding to this perplexity, the absolute necessity of Sec3p is questionable, even though it represents a landmark exocyst complex subunit. At lower temperatures yeast cells can survive if *SEC3* is deleted, suggesting that Sec3p affects the efficiency of vesicle docking whereas Exo70p is the essential effector for exocyst complex tethering [31, 118, 131, 151]. Regardless, the interactions between Sec3p, Exo70p, and the clustering of Rho GTPases and PI(4,5)P_2_ at the PM, initiate the final structural changes necessary for vesicle/PM tethering [132, 138, 142].

Successful vesicle tethering to the PM leads to SNARE-mediated membrane fusion, which is cooperatively facilitated by specific exocyst complex subunits, SNAREs, alongside other regulatory factors. In yeast, the tethering to fusion transition is partially mediated by interactions between Sec6p and Sec3p with the vesicle-bound SNAREs Snc1/2p and the PM target-SNAREs, Sec9p and Sso1/2p [116, 152–155] (Fig. 1C).  Furthermore, the interactions of Sro7p/Sro77p with Sec4p, Exo84p and the t-SNARE Sec9p act in parallel to successfully integrate vesicle tethering and fusion with the PM [118, 139, 140, 156–158]. Functional interactions between *SEC9* and *MSS4* suggest that PI(4,5)P_2_ impacts SNARE-mediated membrane fusion during polarized exocytosis, though it is unclear how [3, 121, 159]. It should be noted that during the specialized vesicular fusion generating prospore membranes during yeast sporulation, PI(4,5)P_2_ binding appears to drive Sso1p (but not Sso2p) conformation changes that specifically activates Sso1p assembly with other SNAREs [160]. However, as a general mechanism, the role of phosphoinositides in membrane fusion during yeast polarized exocytosis remains an open area for future study.

## Budding yeast as a simple model for understanding the impact of phosphoinositide regulation during polarized exocytosis in human diseases

Molecular mechanisms determined from analyzing phosphoinositides in *S. cerevisiae* have direct implications for the therapeutic targeting of polarized exocytosis during host invasion by pathogenic fungi. Non-pathogenic filamentous fungi such as Neurospora species and opportunistic pathogenic yeast such as *Candida albicans* can grow by polarized extensions of hyphae, which represent expansions of the PM supplied by a constant transport of secretory vesicles from the Golgi [161]. In *C. albicans*, Golgi Pik1p-generated PI4P is necessary for the transition to filamentous growth due to its requirement for vesicle biogenesis and targeting to sites of growth at the PM [162]. Unlike *S. cerevisiae*, however, in *C. albicans* the PM synthesis of PI4P by the Stt4p kinase is not essential for cell viability, but the PM PI4P pool is still important for hyphal morphogenesis (just not vesicle trafficking per se) [163, 164]. Although not specifically tested in the context of polarized exocytosis, *C. albicans* Mss4p is localized at filament tips where it increases the localized concentration of PI(4,5)P_2_, which is necessary for hyphal formation and virulence [165, 166]. However, because the PM pool of PI(4,5)P_2_ in *C. albicans* appears normal in the absence of PM Stt4p-generated PI4P, it has been proposed that Golgi PI4P still reaches the PM even though Golgi and PM PI4P pools are separate and distinct [162–164]. Similar to what is hypothesized in *S. cerevisiae*, perhaps *C. albicans* Osh proteins also transfer PI4P from vesicles to the PM for rapid PI(4,5)P_2_ conversion by Mss4p at the PM. If so, *C. albicans* Osh proteins might be efficacious targets for blocking hyphal growth through the inhibition of PI4P transfer to the PM. As small molecule inhibitors of ORPs, and potentially of *C. albicans* Osh proteins, ORPphilins might be effective in disrupting PM phosphoinositide metabolism to prevent hyphal infection [167].

The phosphoinositide-dependence of hyphal growth is pertinent to both pathogenic as well as non-pathogenic fungi, suggesting that PI4P and PI(4,5)P_2_ might be ubiquitously important for polarized exocytosis during filamentation. For instance, the *MSS4* homologue in *Neurospora crassa* is also necessary for hyphal filamentation, and PI(4,5)P_2_ synthesis at the PM is important for polarized membrane growth [168]. The general requirement of PM PI(4,5)P_2_ during fungal hyphal morphogenesis appears to involve coordinating exocytic transport to control the balance between rapid hyphal elongation and the maintenance of polarity at hyphal tips [169, 170]. Although well conserved from fungi to humans, PI transfer proteins with homology to *S. cerevisiae* Sec14p offer another target for inhibiting fungal Golgi-synthesized PI4P to block virulence factor secretion and the transition to filamentous growth [171–173]. It has been proposed that structural subtleties in Sec14p homologues can be exploited to develop small molecular inhibitors with binding selectivities permitting effective antifungal activity without off-target toxicity [174, 175].

As mechanistically defined in *S. cerevisiae*, the conserved exocyst complex and its regulation via interactions with phosphoinositides might also contribute to polarized exocytosis necessary for hyphal formation in filamentous fungi (Table 1) [165, 176–180]. Unlike *S. cerevisiae*, however, polarized exocytosis to hyphal growths on the PM proceeds through a subapical intermediate of clustered Sec4p-associated vesicles called the Spitzenkörper [181, 182]. Although the Spitzenkörper does not confer directionality to vesicle trafficking per se, subsequent exocyst complex targeting of secretory transport supports the polarized growth of hyphae at the PM [176, 177, 183, 184]. In terms of potential phosphoinositide-binding proteins, the Sec3p homologue in Candida is required for hyphal formation, as is the Sec2p homologue [185, 186]. At the PM, Rho GTPases is generally required for polarized membrane growth of hyphal filaments, which is partly affected by phosphoinositides [187–191]. In Candida, however, the polarized localization of Cdc42p is independent of PI(4,5)P_2_, but active Rho1p recruitment to growing hyphal filament is dependent on Stt4p and Mss4p synthesis of PI4P and PI(4,5)P_2_ at the PM [190]. If mechanistically akin to exocyst complex tethering in *S. cerevisiae*, phosphoinositide regulation of PM Rho family GTPases is likely an important determinant of polarized exocytosis during hyphal filamentation.

**Table 1:** Yeast, filamentous fungi, and mammalian polarized exocytosis regulators, effectors, and interactions with PI4P and/or P(4,5)P_2_ as linked to human disease

Yeast protein	Phosphoinositide bound	Binding mechanism	Localization	Activity	Mammalian homologue(s)	Filamentous fungi homologue(s)	Associated human disease(s)	References
Sac1	PI4P	Sac domain	ER	PI4P 4-phosphatase	SACM1L	Sac1^a^	N/A	[22, 162]
Ors2	PI4P	Split PH domain	Golgi	PS/PC P4-type ATPase (flippase)	ATP8A1/2	Drs2^a^	Cerebellar Ataxia and Dysequilibrium Syndrome 1 and 4	[88, 243–245]
Bet3 (TRAPPII subunit)	PI4P	Polybasic cluster	Golgi	Rab GEF	TRAPPC3, TRAPPC3L		N/A	[246]
Sec14	PI4P	Pocket	Golgi	PI/PC Transport	Sec14L1/2/5/6	Sec14^a^	Breast cancer Prostate cancer	[172, 247, 248]
Osh4	PI4P	Pocket	Golgi/Vesicles/PM	PI4P/Sterol Transport	OSBPL10/11	Osh4^a^	N/A	[44, 245]
Myo2	PI4P	COOH-terminal tail	Vesicles	Vesicle motor	MYO5C/A	Myo2^a^	Griscelli Syndrome Type 1 and 3	[110, 249–252]
Sec2	PI4P	Polybasic loops	Vesicles	Rab GEF	RAB3IP, RAB3IL1, Rabin8	Sec2^a^	Parkinson’s disease	[108, 229]
Exo84	PI(4,5)P2	Polybasic cluster	Vesicles/PM	Vesicle Tethering	EXOC8	Exo84^a^, EXO-84^b^	Colorectal cancer Joubert syndrome Neurodevelopment Disorder Skin squamous cell carcinoma	[184, 223, 253–258]
Exo70	PI(4,5)P2	Polybasic cluster	Vesicles/PM	Vesicle Tethering	EXOC7	Exo70^a^, EXO-70^b^, *Agexo70*^c^	Microcephaly	[30, 135, 176, 181, 184, 212, 253, 257]
Sec3	PI(4,5)P2	PH domain	Vesicles/PM	Vesicle Tethering	EXOC1	Sec3^a^, SEC3^b^, *Agsec3*^c^	N/A	[31, 135, 176, 184, 185]
Cdc24	PI(4,5)P2	PH domain	PM	Rho GEF	PLEKHG4B, KALRN	Cdc24^a^	Coronary artery disease, Cerebral atherosclerosis	[128, 259, 260]
Rom1/2	PI(4,5)P2	PH domain	PM	Rho GEF	NET1		Neuroepithelioma breast cancer	[261–263]
Mss4	PI4P	Kinase domain	PM	PI4P-5-Kinase	PIP5K1A/BC, PIP4K2A, PIP5K2A	Mss4^a^, MSS-4^b^	Bipolar disorder, cancer, fetal neural defects, Friedreich Ataxia, Lethal Lymphoblastic leukemia, schizophrenia	[165, 168, 204–206, 264, 265]
Gic1/2	PI(4,5)P2	Poly basic cluster	PM	Cdc42 effector	N/A		N/A	[130]
Inp51-Inp54	PI(4,5)P2	Sac domain	ER, PM, endosomal compartments	Inositol polyphosphate 5-phosphatase	OCRL		Lowe’s Syndrome Dent Disease 1 and 2	[198, 266–268]

^a^Candida albicans

^b^Neurospora crassa

^c^Ashbya gossypii

Apart from hyphal formation and fungal pathogenesis, the regulation of phosphoinositide metabolism during polarized exocytosis can impact human disease, which in some cases can be intuited through the conserved mechanisms as originally defined in *S. cerevisiae*. Defects in phosphoinositide metabolism and regulation are associated with specific cancers, neuromuscular disease and neurodegenerative disorders, including Parkinson’s disease and amyotrophic lateral sclerosis (ALS) [192, 193] (Table 1). While phosphoinositide dysregulation has been directly linked to disorders affecting endolysosomal trafficking or synaptic vesicle exocytosis, few diseases associated with defective polarized exocytosis can be directly traced to changes in phosphoinositide metabolism. For example, metastatic loss of cell–cell adhesion and cell migration is promoted by increased Golgi PI4P, which is otherwise attenuated by the Sac1 inositol 4-phosphatase [22, 194]. However, Sac1 itself has yet to be directly linked to a specific human disease, let alone to one caused by polarized exocytosis defects [195]. In contrast, the dual inositol 4- and 5-phosphatases represented by the Sac1 domain-containing synaptojanins affect other membrane trafficking pathways directly impacting disease [192, 193, 195]. Mutations in the Synaptojanin-1 Sac1-domain disrupts endocytosis and cilia regulation and causes early-onset Parkinson’s disease [196, 197]. Although it lacks a Sac1-domain, OCRL is another inositol 5-phosphatase that hydrolyzes PI(4,5)P_2_ in the ciliary axoneme, and is linked to a ciliopathy causing Lowe’s syndrome [198] (Table 1). OCRL teams up with Sac2/INPP5F, a Sac1-domain containing inositol 4-phosphatase, to dephosphorylate PI(4,5)P_2_ akin to the dual phosphatase activities of synaptojanins (and in mice Sac2/INPP5F knock-out synergistically aggravates Synaptojannin-1-related Parkinson’s disease defects) [196, 199]. Because endocytosis and cilia regulation require PI(4,5)P_2_ dephosphorylation at the PM, whereas polarized exocytosis requires local accumulations of PI(4,5)P_2,_ synaptojanin inactivation might even bolster PI(4,5)P_2_-dependent polarized exocytosis. Indeed, the deletion of yeast Sac1-domain synaptojanins encoded by *INP51*-*INP53* perturbs endocytosis, but not exocytosis; though synaptojanin overexpression might perturb polarized exocytosis by reducing PM PI(4,5)P_2_ [1]. In the specific cases of synaptic vesicle trafficking and regulated exocytosis, the generation of both PI4P and PI(4,5)P_2_ have important functions that impact the pathology of neurodegenerative disease and mental disorders [200–203]. However, in diseases affecting polarized membrane trafficking, a direct mechanistic link for many PI4P and PI(4,5)P_2_ kinases and phosphatases is less established. In yeast, overexpression of the Mss4p PI4P 5-kinase rescues defects in polarized exocytosis caused by mutations in *CDC42*, *MYO2*, and genes encoding exocyst subunits [3, 121]. Human homologues of Mss4p (PIP5K1C and PIP4K2A/PIP5K2A) are implicated in fetal neural/arthrogryposis defects and schizophrenia, respectively [204–206] (Table 1). In terms of PI4P generation, defects in human PI 4-kinase (PI4K) IIIα are associated with the neurological disease hypomyelinating leukodystrophy [207, 208]. In flies, PI4K IIIα defects disrupts the recruitment of the exocyst complex subunit Sec5 [37]. Although it is unclear whether polarized exocytosis is specifically compromised in human phosphoinositide kinase-associated diseases, membrane trafficking to the PM might be an aggravating factor. Analogous to yeast, activation of phosphoinositide kinases might also rescue disease-causing defects in human exocyst complex subunits and regulators. Of the many pharmaceutical targets of PI4P and PI(4,5)P_2_ signaling, key effector complexes of polarized exocytosis might represent opportunities for therapeutic manipulation [209].

The mammalian exocyst complex drives selective secretion to polarized sites on the PM [210–212], but the exocyst complex also mediates constitutive exocytosis in mammals [213]. As such, the exocyst complex is required for neurite outgrowth during neuronal development and membrane dynamics, despite that the exocyst complex is not involved in synaptic transport and neurotransmitter release [214–216]. The exocyst complex is important for targeting secretory cargo to cilia, which affects the cellular detection of sensation and extracellular signaling [217, 218]. Mutations in many exocyst complex components also inhibit epithelial polarization in mammalian cells by disrupting transport desmosomes and adherens junctions [219, 220]. As a result, defects in specific components in the exocyst complex are causative agents of neurodegeneration and ciliopathies [211, 212] (Table 1). Defects in the human homologues of yeast *SEC5* (corresponding to human *EXOC2*), *SEC6* (*EXOC3L2*), *SEC8* (*EXOC4*), *SEC15* (*EXOC6B*), *EXO70* (*EXOC7*), *EXO84* (*EXOC8*), and *RHO3* (*RALA*) are associated with a variety of neurodegenerative and neurodevelopmental disorders [212]. Given the mechanistic conservation between yeast and humans, it is not surprising that many mutations that disrupt yeast polarized exocytosis cause related defects in humans manifesting in disease.

As with the yeast exocyst complex, the corresponding metazoan components and effectors share a similar dependence on phosphoinositide regulation. Like in yeast, the interaction between PI(4,5)P_2_ and the mammalian Exo70 homologue (ExoC7) confers PM association [30, 221, 222]. In addition to ExoC7, several mammalian and yeast exocyst subunits contain phosphoinositide-binding pleckstrin homology (PH) domains or other PI4P/PI(4,5)P_2_ interacting motifs (Table 1). Mutations in these domains and motifs can disrupt binding to phosphoinositides and can have disease-related consequences. A patient afflicted with Joubert syndrome ciliopathy is homozygous for a mutation within the phosphoinositide-binding PH domain of ExoC8, underscoring the disease relevance of exocyst complex-phosphoinositide interactions [223]. This is consistent with the consequence of losing all EXOC6B function, which results in ciliopathy that causes nervous system and joint impairment [224]. Although not directly linked to any named diseases, mammalian *EXOC5* (corresponding to yeast *SEC10*) and Cdc42 cooperatively interact in the same pathway during ciliogenesis [225–227]. In yeast, *SEC10* mutations are rescued by overexpression of the PIP4 5-kinase encoded by *MSS4*, and Cdc42p localization to the PM is dependent on Mss4p function [3, 121]. Thus, based on the yeast findings, the generation of PI(4,5)P_2_ might also affect the regulation of the ciliogenesis pathway by *EXOC5* and Cdc42. Another significant factor in ciliogenesis regulation involves the human homologue of *SEC2*, encoded by Rabin8 [228]. Rabin8 activation of the Rab8 GTPase (the human homologue of yeast Sec4p) is blocked by PTEN-induced kinase 1 (PINK1), an autosomal recessive agent of Parkinson’s disease [229], suggesting a link between Parkinson’s disease and Rab8 activation by Rabin8. If the yeast Sec4p-Sec2p regulatory mechanism applies to the Rab8-Rabin8 interaction, then vesicular PI4P might impact the progression of Parkinson’s disease. The complex interactions between exocyst complex subunits, their regulators, and both PI4P and PI(4,5)P_2_ provides therapeutic opportunities for neurodegeneration and ciliopathies. In addition, cancer is also affected by polarized PM trafficking that might be treated via phosphoinositides.

Because the exocyst complex impacts polarized membrane growth, and cancer is often driven by changes in cell polarization, recent findings are consistent with the predication that the exocyst complex directly impacts tumorigenesis [230] (Table 1). For example, knockdown of *EXOC2* or *EXOC8* inhibits Ras-dependent tumorigenic human cell growth. ExoC2 and ExoC8 act as direct effectors of RalA and RalB GTPases, which in turn are Ras effectors liked to several cancers [231–233]. Although Ral GTPases are not present in yeast [146, 234], *CDC42* and *RHO3* Rho family GTPases functionally interact with yeast *SEC5* and *EXO84*, suggesting a loose mechanistic conservation [118, 119, 139, 235]. In humans, RalA interactions are thought to represent an alternate pathway for exocyst complex activation causing vesicle clustering near the PM, which is predicted to boost subsequent membrane fusion events [118, 146]. In this regard, the RalA GTPase interaction with ExoC8 (i.e. human *EXO84*) has been proposed to functionally emulate the interaction of Sro7p with Exo84p in yeast [118, 145]. Furthermore, phosphoinositides appear to regulate the Ral GTPase-ExoC8 interaction [146]. The Ral GTPase binding site overlaps with the ExoC8 PH domain, and RalA competes with phosphoinositides for ExoC8 binding. The human homologue of yeast Sro7p, human lethal (2) giant larvae (Hugl), affects polarized exocytosis and is directly implicated in tumorigenesis [156, 236], suggesting that the yeast Sro7p-Exo84p interaction might also have implications for cancer. Because of the physical and functional interconnectivity of all exocyst complex subunits, targeted changes in PI4P or PI(4,5)P_2_ binding or phosphoinositide metabolism might offer new treatment options for tackling tumorigenesis [209].

## Conclusions

The effects of phosphoinositide signaling during polarized exocytosis are focused on specific regulators that mediate transitions between mechanistic steps. Phosphoinositides help drive these transitions by promoting the switching of one small GTPase for the activity of another in the regulatory cascade that defines the overall process of exocytosis [17]. Vesicle biogenesis is dependent on PI4P induction of the Arf1p GTPase and then, in preparation for membrane tethering to the PM, exocyst assembly requires PI4P removal from post-Golgi vesicles for Sec2p GEF activation of the Sec4p GTPase. In parallel, the targeting of vesicles to sites of membrane growth on the PM is dictated by Cdc42p and Rho GTPase polarization by PI(4,5)P_2_. PI(4,5)P_2_ further supports exocyst complex interactions with the PM through direct interactions principally with Exo70p. The control of each phosphoinositide regulatory node is conferred by the dynamic actions of lipid transfer proteins (i.e. Sec14p and Osh4p) acting in concert with agents of PI4P and PI(4,5)P_2_ metabolism (i.e. Sac1p, Stt4p, Pik1p, and Mss4p).

During Golgi vesicle biogenesis, Arf1p activation is intimately coupled to PI4P metabolism and vice versa. PI4P synthesis by Pik1p leads to Ypt31p/32p GTPase recruitment that induces the Arf1p GEF, Sec7p (Fig. 2). PI4P synthesis by Pik1p is further increased by Arf1p activation, which ultimately drives a positive feedback loop for ensuring Arf1p completion of vesicle release [77]. The mechanism controlling this Arf1p feedback loop is less understood. PI4P synthesis is kept in check by Osh4p and Sac1p turn-over of PI4P, and deletion of *OSH4* and *SAC1* bypasses the essential requirement for Sec14p, just like eliminating the Arf1 GAPs, Age1p and Gcs1p [79]. Gcs1p also binds PI4P in vitro (though Gcs1p has a general affinity for monophosphorylated phosphoinositides) [237], which suggests that Gcs1p might be recruited to the Golgi when high PI4P levels to break the Arf1p positive feedback loop. In addition, Age2p contains a C-terminal domain amphipathic helix that mediates Age2p binding to membranes in vitro [238]. It is a reasonable prediction that PI4P promotes Age2p membrane interactions via this region, in a manner akin to Gcs1p-PI4P binding.

Given its central importance in driving the mechanistic transition from vesicle biogenesis to vesicle targeting, Sec2p demands special consideration. During the transit of post-Golgi vesicles to the PM, PI4P acts directly on Sec2p to dictate when the Sec4p GTPase is activated and when Sec15p binding initiates exocyst complex assembly. The dynamics of PI4P exchange on post-Golgi vesicles is therefore key to changing Sec2p activity states. The depletion of PI4P from vesicle membranes by Osh4p and other yeast Osh proteins represents an important trigger for vesicle docking. It remains an open question as to what controls the timing of PI4P removal by Osh proteins and, ultimately, where this PI4P is transferred.

Whereas PI4P is the primary phosphoinositide impacting vesicle regulation, PI(4,5)P_2_ in the PM determines where vesicles will dock. The contribution of PI(4,5)P_2_ to Cdc42p polarization and, by extension, to polarized exocytosis has been enigmatic. Initial Cdc42p polarization is conferred in part by its GEF Cdc24p, which in turn seems to be polarized through cooperative binding to anionic lipids via a PH domain that has very low affinity for phosphoinositides [128]. Once the polarity axis is established, the Cdc24p-Cdc42p interaction is PI(4,5)P_2_-independent, but other interactions that maintain Cdc42p polarization are affected by PI(4,5)P_2_ [3]. Several Cdc42p and Rho GTPase effectors/regulators have PH domains or polybasic domains that exhibit high affinity for PI(4,5)P_2_ [130, 239, 240]. In addition to Exo70p and Sec3p binding to PI(4,5)P_2_, the polarized concentration of PI(4,5)P_2_ appears to be essential to the maintenance of cell polarization, even if not critical for its establishment. Although PI(4,5)P_2_ diffusion appears to be reduced in regions with high densities of phosphoinositide-binding effectors, PI(4,5)P_2_ has limited ability to organize into membrane domains [35, 241]. The lack of intrinsic mechanisms for PI(4,5)P_2_ clustering presents a chicken-and-egg scenario with respect to the role PM polarization. A dynamic equilibrium of PI(4,5)P_2_ synthesis and phosphoinositide transfer might offer a solution to the paradox of how a freely mobile lipid is seemingly retained and localized to one membrane site. Indeed, overexpression of Osh4p increases Cdc42p localization to the PM at sites of polarization, consistent with the notion that Osh-dependent transfer of PI4P to the PM concentrates PI(4,5)P_2_ to maintain Cdc42p polarization [48].

In general, the phosphoinositide interaction nodes represented by Arf1p, Sec2p-Sec4p (i.e. Rab8-Rabin8), and Cdc42p might represent optimal therapeutic targets for polarized exocytosis-related disease. As one approach, structure-based design can be used to develop allosteric inhibitors and activators specific for effector PH domains. If Rabin8 shares the same lipid affinities, PI4P binding pockets are viable targets to control its dysfunction in ciliopathies and potentially in PINK-dependent Parkinson’s. More generally, phosphoinositide kinases and phosphatases, as well as phosphoinositide transfer proteins, also represent druggable factors to curb disruptions in polarized exocytosis that induce disease.

## Data Availability

All data of this study are available within the article.
